# Genome-wide detection and characterization of positive selection in Korean Native Black Pig from Jeju Island

**DOI:** 10.1186/s12863-014-0160-1

**Published:** 2015-01-30

**Authors:** Jaemin Kim, Seoae Cho, Kelsey Caetano-Anolles, Heebal Kim, Youn-Chul Ryu

**Affiliations:** Interdisciplinary Program in Bioinformatics, Seoul National University, Seoul, 151-742 Korea; CHO&KIM genomics, Main Bldg. #514, SNU Research Park, Seoul National University Mt.4-2, NakSeoungDae, Gwanakgu Seoul, 151-919 Republic of Korea; Department of Agricultural Biotechnology and Research Institute of Population Genomics, Seoul National University, Seoul, 151-742 Republic of Korea; Department of Animal Sciences, University of Illinois, Urbana, IL 61801 USA; Division of Biotechnology, The Research Institute for Subtropical Agriculture and Biotechnology, Jeju National University, Jeju, 690-756 Republic of Korea

**Keywords:** Korean native black pig, Jeju black pig, Positive selection

## Abstract

**Background:**

In the 1980s, Korean native black pigs from Jeju Island (Jeju black pigs) served as representative sample of Korean native black pigs, and efforts were made to help the species rebound from the brink of extinction, which occurred as a result of the introduction of Western pig breeds. Geographical separation of Jeju Island from the Korean peninsula has allowed Jeju black pigs not only to acquire unique characteristics but also to retain merits of rare Korean native black pigs.

**Results:**

To further analyze the Jeju black pig genome, we performed whole-genome re-sequencing (average read depth of 14×) of 8 Jeju black pig and 6 Korean pigs (which live on the Korean peninsula) to compare and identify putative signatures of positive selection in Jeju black pig, the true and pure Korean native black pigs. The candidate genes potentially under positive selection in Jeju black pig support previous reports of high marbling score, rare occurrence of pale, soft, exudative (PSE) meat, but low growth rate and carcass weight compared to Western breeds.

**Conclusions:**

Several candidate genes potentially under positive selection were involved in fatty acid transport and may have contributed to the unique characteristics of meat quality in JBP. Jeju black pigs can offer a unique opportunity to investigate the true genetic resource of once endangered Korean native black pigs. Further genome-wide analyses of Jeju black pigs on a larger population scale are required in order to define a conservation strategy and improvement of native pig resources.

**Electronic supplementary material:**

The online version of this article (doi:10.1186/s12863-014-0160-1) contains supplementary material, which is available to authorized users.

## Background

The Korean native black pig (KNBP) represents only a minor proportion of the total pig population in Korea, yet the demand for its meat product is exceptionally high due to its higher fat content and redness compared to that of other commercial breeds [1]. Although the economic value of this breed is well appreciated, KNBP shows a relatively slower growth rate and lighter carcass weight [2], which has led to the introduction of improved breeds such as Hampshire and Berkshire pigs for both growth and lean meat production since the 1970’s [3]. This massive influx of industrial pig breeds has resulted in a significant recession in the population of native pig, as well as a loss of genetic resources. KNBP has been reported to comprise only around 0.74% of a total of 9.19 million pigs in Korea [1]; most black pigs in Korea appear to be the crossbreds of untraceable origin [4]. The National Livestock Research Institute in Korea [5] selected Korean native black pigs from Jeju Island (or Jeju black pig, JBP) as a representative sample of KNBP and began attempts to restore and conserve genetic diversity of the native pig species in 1988. JBP has been isolated from the main Korean peninsula, and this long-term isolation has resulted in unique genetic characteristics of the JBP in addition to its inherent characteristics as KNBP.

JBP is considered as the rare representative of true KNBP [4], of which genetic resources are of prime importance in industrial breeding programs. JBP is known for higher marbling score than Western breeds [6] and desirable characteristics such as tenderness, juiciness, redness and brightness [2], besides its strong disease tolerance [1]. It is also known that JBP rarely showed PSE (pale, soft, exudative) appearance [2], where PSE describes a carcass quality condition characterized by the dry meat and unattractive to consumers. However, the biological basis for these characteristics of JBP has not been clearly demonstrated.

Recently, several studies have identified loci under selection to unveil the selective pressures at the genomic level to identify candidate genes associated with economic traits in pigs [7]. For example, Li et al. identified the *MC1R* gene which has a key role to black coat color in Chinese domestic pigs from selection signatures [8]. Rubin et al. searched for genetic variants showing allele frequency differences between pig and wild boar populations to reveal some genomic regions that underlie phenotypic evolution in European domestic pigs [9].

To better understand the genome-wide genetic structure of JBP population and search for signatures of positive selection, the whole genomes of 8 Jeju JBP and 6 KP were sequenced. As mentioned earlier, most pigs in Korea (KP) have been crossed with European pig breeds and thus are not true representatives of Korean native black pigs. Using KP as a comparable population to JBP, we applied haplotype test to decipher regions under positive selection in JBP of which genetic resources help understand KNBP that are gradually rebounding from the verge of extinction.

## Methods

### Samples and DNA re-sequencing data

Whole-blood samples (10 mL) were collected from 8 JBP and 6 KP according to the guidelines for the Care and Use of Laboratory Animals of the Institutional Ethical Committee of Jeju National University. Paired-end reads were generated using Illumina HiSeq2000. DNA was extracted from whole blood using a G-DEXTMIIb Genomic DNA Extraction Kit (iNtRoN Biotechnology, Seoul, Korea). 3 μg of genomic DNA was randomly sheared using the Covaris System to generate inserts of ~300 bp. Using the TruSeq DNA Sample Preparation Kit, the DNA fragments were end-repaired, A-tailed, adaptor ligated, and amplified. Paired-end sequencing was performed by NICEM (National Instrumentation Center for Environmental Management of Seoul National University) using the Illumina HiSeq2000 platform with TruSeq SBS Kit v3-HS (Illumina). Finally, sequence data was generated using the Illumina HiSeq system.

The paired-end reads were then mapped against the Sus scrofa reference genome (Sscrofa 10.2) using Bowtie2 [10]. We used default parameters (except the “–no-mixed” option) to eliminate unpaired alignments for paired reads. An average read depth of 14.26× (9.89× ~ 16.98×) was achieved, and on average across all samples, the reads covered 98.60% of the genome (Additional file 1: Table S1).

Several open-source software packages were used for downstream analyses and variant calling. Adopting the “REMOVE_DUPLICATES = true” option in the “MarkDuplicates” command-line tool of Picard (http://picard.sourceforge.net), potential PCR duplicates were excluded. We then used SAMtools [11] to construct index files for reference and bam files. Relying on the arguments such as “RealignerTargetCreator” and “IndelRealigner” arguments, genome analysis toolkit 1.4 (GATK) [12] was used to perform local realignment of reads to correct misalignments due to the presence of insertions/deletions.

Further, the “UnifiedGenotyper” and “SelectVariants” arguments of GATK were used for identifying candidate SNPs. In order to minimize possible false positives, argument “VariantFiltration” of the same software was used to filter variants with the following criteria: 1) phred-scaled quality score < 30; 2) MQ0 (mapping quality zero, which is total count across all samples of mapping quality zero reads) > 4 and quality depth (unfiltered depth of non-reference samples; low scores are indicative of false positives and artifacts) < 5; and FS (Phred-scaled P-value using Fisher’s exact test, which represents variation on either the forward or the reverse strand, which are indicative of false positive calls) >  200.

BEAGLE was used [13] to infer the haplotype phase for the entire set of pig populations. A summary of the total number of SNPs and a distribution plot of SNPs along the genome are provided in Additional file 1: Table S2 and Figure S1.

### Detection of genomic regions with putative signals of selection

Using whole SNP sets defined from both JBP and KP, the method cross-population extended haplotype homozygosity (XP-EHH) was used to detect genome-wide selective sweep regions (http://hgdp.uchicago.edu/Software/) [14]. XP-EHH defines two populations (A and B), a core SNP, and a SNP X that are up to 1 Mb from the given core SNP. A SNP X is selected such that its EHH with respect to all chromosomes in both populations is as close as possible to 0.04. Next, the test focuses on the chromosomes in each population to calculate EHH at all SNPs between the core SNP and X; integrates it within these bounds (results are called IA and IB, respectively); finally defines an XP-EHH log-ratio as ln(IA/IB) [15]. An XP-EHH score is directional: an extreme positive score implies selection in JBP, while a negative score suggests selection in the KP population. The log ratios were standardized to have a mean of 0 and variance of 1. An XP-EHH raw score distribution plot is provided in Additional file 1: Figure S2. We then split the genome into non-overlapping segments of 50 kb to use the maximum XP-EHH score of all SNPs within a window producing a summary statistic for each window. To consider the SNP frequency, genomic windows were binned based on their numbers of SNPs in increments of 200 SNPs (combining all windows with more than 600 SNPs into one bin). Within each bin, for each window *j*, the fraction of windows with a value of the statistic greater than that in *j* is defined as the empirical P-value, according to the method previously introduced [15,16]. The regions with P-values less than 0.01 (1%) were considered strong signals in JBP. Throughout the paper, the “P-values” indicate empirical P-values; in other words, a low P-value implies that a locus is an outlier with respect to the rest of the genome. As the loss of power incurred by decreasing sample size is known to be modest with 20 chromosomes when size of second population is fixed [15], minimum power loss in our study (16 JBP) can be expected.

Additionally, the cross-population composite likelihood ratio test (XP-CLR) for detecting selective sweeps that involves jointly modeling the multilocus allele frequency between two populations were performed [17]. XP-CLR scores were calculated using scripts available at (http://genetics.med.harvard.edu/reich/Reich_Lab/Software.html). The following parameters were used: non-overlapping sliding windows of 50 kb, maximum number of SNPs allowed within each window as 400, and correlation level of 0.95 to down-weight the pairs of SNPs in high LD. The regions with the XP-CLR values in the top 1% of the empirical distribution (XP-CLR > 79.39) were designated candidate sweeps.

### Minor allele frequency analysis and Tajima’s D statistic

For each population, the minor allele frequency (MAF) was calculated at every position using VCFtools 4.0 [18]. The distribution of MAF along the genome is provided in Additional file 1: Figure S3. The proportion of SNPs with allele frequencies lower than threshold (MAF < 0.10) was then calculated within sliding windows of 100 kb in size every 20 kb, comprising a total of 127,888 bins. This threshold was chosen to maximize sensitivity as suggested by previous studies [19,20], and we also applied a minimum number of SNPs per window (at least 10 SNPs). Tajima’s D was calculated in bins with size 50 kb using the Arlequin software [21]. The significance was determined by performing coalescent simulation. The probability distribution of Tajima’s D under neutrality was generated by 10,000 random samples under the assumption of selective neutrality. The genomic regions were considered significant where P(D_simul_ < D_obs_) < 0.05. The resulted line was smoothed using the function *lowess* in the R package.

### Population structure analyses

Genotype data was restricted to a random subset of ~1% (159,660 SNPs) of total SNPs using PLINK (-thin option) [22]. The population structure of JBP and KP was analyzed using STRUCTURE version 2.3. [23]; the “admixture” model was run with K = 2 and 20,000 iterations after a burn-in of 100,000 iterations was selected.

### Linkage disequilibrium (LD) and Haploview analysis

On genotype data for 159,660 randomly selected SNPs, genome-wide LD was estimated by calculating the squared correlation coefficient (*r*^*2*^) between all pairs of SNPs with inter-SNP distances of less than 10 Mb both within a given breed using PLINK (r2 and ld-window options) [22]. Observed pair-wise LD was averaged for each 50-kb inter-SNP distance bin. The software Haploview was used to calculate pairwise measures of linkage disequilibrium (LD) among SNPs within candidate gene regions and to create a visual representation of data [24].

### Characterization of candidate genes under selection

“Significant” genomic regions identified from XP-EHH and XP-CLR tests were annotated to the closest genes (Sscrofa 10.2). Genes that spanned (partially or completely) the window regions were defined as candidate genes. Gene and pathway analyses was performed using DAVID (Database for Annotation, Visualization and Integrated Discovery) [25]. Positively selected genes were functionally explored and visualized by gene ontology using the ClueGo plugin of Cytoscape [26,27].

## Results and discussion

### Sequencing, assembly and identification of SNPs

The genomes of 8 JBP and 6 KP were sequenced to 14.26× coverage on average, with a total of reads comprising ~492 Gbp. Using Bowtie 2 [10], reads were aligned to the reference pig genome sequence (Sscrofa 10.2) to cover 98.60% of the genome (Additional file 1: Table S1). After filtering potential PCR duplicates and correcting for misalignments due to the presence of INDELs, we detected SNPs using GATK [28]. We then removed SNPs to lower the false positives based on the following criteria: phred-scaled quality score, mapping quality, quality depth and phred scaled P-value. We finally retained a total of ~15.91 million (M) SNPs, comparable to recent studies of 18.68 M, 9.49 M and 6.79 M SNPs identified from diverse pig breeds [9,29,30] (Additional file 1: Table S2).

### Population structure and extent of linkage disequilibrium

**Figure 1:**
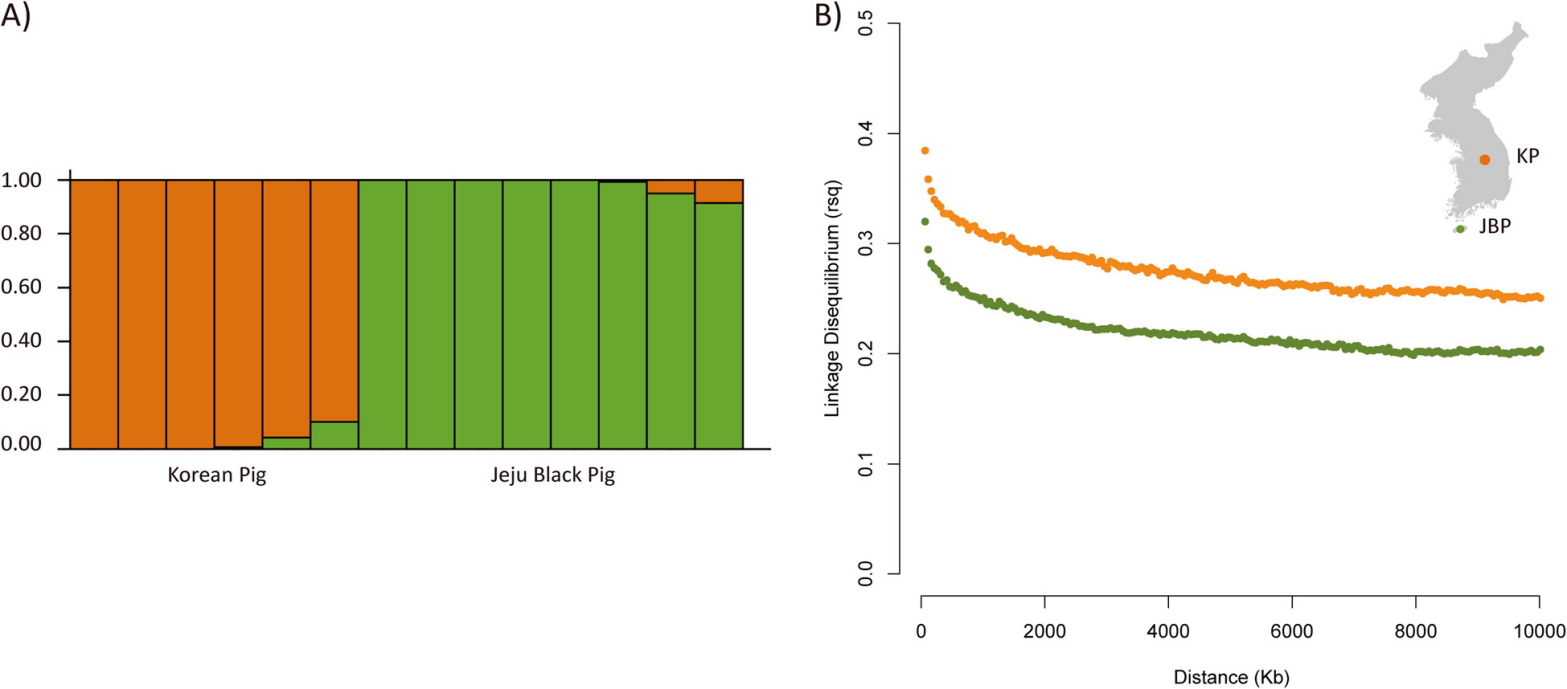
**Estimated population structure and LD decay in Jeju black pig and Korean pig breeds. (A)** The proportion of ancestry for each individual in K = 2 is shown. Colors in each vertical line show the likelihood to which source population an individual can be assigned. **(B)** Genome-wide linkage disequilibrium was estimated in each breed, by calculating r2 values between all pairs of SNPs with inter-SNP distances less than 10 Mb.

We investigated the genetic structure using a Bayesian approach to infer population structure between two breeds on a random subset of 159,660 SNPs [23]. Assuming two source populations (K = 2), the program assigns all individuals to either JBP or KP (Figure 1A). This genetic clustering analysis provided no concrete support in favor of population admixture between JBP and KP.

Using a subset of SNPs, genotypes for all SNP pairs less than 10 Mb apart were evaluated to estimate genome-wide linkage disequilibrium (LD) across two breeds. Average r^2^ at various distances in classes of 50 kb was computed by grouping all SNPs combinations. The LD decays with increasing distance for both breeds but also shows discrepancy in strength between two breeds (Figure 1B). SNP pairs at a distance of 0.5 Mb had an average r^2^ of 0.26 for JBP and 0.32 for KP, both of which are closer to that for Chinese breeds than for European breeds, as the European pigs showed a higher level of LD [31]. In addition, a greater extent of LD in KP compared to JBP may show evidence of past introgression from Western breeds, coinciding with the historical background of pig industry in Korea.

### Putative selective signature in Jeju black pig population

Haplotype homozygosity was estimated between the JBP and KP populations using the cross population extended haplotype homozygosity (XP-EHH) algorithm. The XP-EHH statistic estimates haplotype differences between two populations and is designed to detect alleles that have increased in frequency to the point of fixation or near-fixation in one of two populations. The haplotypes that are more frequent and longer than expected arise due to the random processes considered to be positively selected [14,15]. To test the hypothesis that unique characteristics in Jeju black pig is majorly driven by positive selection, we searched for long haplotypes in JBP compared to KP. Sets of regions that showed evidence of local positive selection were identified using an empirical significance level of 0.01. These outlier genomic regions provide specific candidate regions for fine-scale mapping of genes that are important for unique characteristics in JBP. In our study, the test detected a total of 212 JBP putatively advantageous genes (Table 1 and Additional file 2: Table S3).

**Table 1:** **Summary of major genes selected from genome-wide scan (see Additional file** **2** **: Table S3 and Additional file** **3** **: Table S4 for summary values of all candidate genes)**

**Genes identified in 50-kb region**	**CHR** ^**1**^	**Max XP-EHH**	**XP-EHH P-value**	**XP-CLR**	**Tajima’s D (P-value)**
*THRB*	13	-	-	93.73	-
*PPIL6, ZBTB24, SMPD2*	1	-	-	114.20	-
*TLR3*	15	4.54	0.0028	-	-1.55 (P = 0.035)
*ACSL6*	2	4.14	0.0094	84.96	-1.66 (P = 0.025)
*ACE*	12	-	-	122.14	-
*CACNA1I*	5	4.12	0.0100	86.80	-
*ATP6V1H*	4	-	-	90.84	-1.68 (P = 0.029)

^1^chromosome.

Dash (-) indicates non-significant statistics.

If each signature provides distinct information about positive selection, combining signals provides greater power for localizing the source of selection [32]. For this reason, we used the XP-CLR statistic, which evaluates allele frequency differentiation between populations to identify candidate regions for selective sweeps. This statistic is particularly robust to ascertainment bias and population demography. Using the top 1% of the empirical distribution among genomic regions, 251 genes were identified, 71 of which were observed in the intersection of XP-EHH selection candidates, comprising a total of 392 candidate genes under positive selection in JBP (Additional file 3: Table S4).

### Genes responsible for pale, soft, exudative (PSE) meat

Pale, soft, exudative (PSE) pork was first recognized in 1953. The undesirable appearance and texture, limited functionality, and inferior processing yield of PSE pork continued to make it a critical quality and economic concern [33,34]. Rapid postmortem muscle acidification combined with high muscle temperature, as well as low ultimate meat pH have long been implicated as factors that induce PSE pork characteristics [35]. By the 1980s, it was recognized that an abnormal calcium release mechanism was a key factor in the increased frequency of PSE meat [36,37], and the genetic basis of this syndrome was identified as a point mutation in the ryanodine receptors or RyRs [38]. It is known that KNP rarely showed PSE-like appearance [2]. We identified thyroid hormone receptor, *THRB* (XPCLR = 93.73), as a positively selected gene. Thyroid hormones may also alter intracellular Ca2+ homeostasis in skeletal muscle by direct action on RYR to increase the open state probability of the channel, thereby increasing Ca^2+^ flux [39]. The previous studies thus suggested that an aberrant thyroid hormone response to heat stress may occur in stress-susceptible as well as growth-selected animals, which might lead to the abnormality of Ca^2+^ regulation and thus subject animals to the development of PSE meat [40]. Seven genes (*FKBP1B*, *JAK2*, *CD24*, *PTK2B*, *CACNA1I*, *CCR7*, *EPHX2*) involved in calcium ion homeostasis (GO: 0055074) were also positively selected in JBP.

### Genes indicative of positive selection that are potentially related to JBP meat quality

Fatty acids are involved in various “technological” aspects of meat quality. Variation in fatty acid composition leads to different melting points and thus influences on the firmness or softness of the fat in meat, especially the subcutaneous, intermuscular (carcass fats) and the intramuscular (marbling) fat [41]. JBP are known for a high content of unsaturated fatty acid which contributes to the better meat quality. Therefore, we investigated genes involved in fatty acid composition based on its gene function and gene ontology. Gene ontology analysis revealed *CD36* (P = 0.0036; XP-EHH = 4.67) and *ACE* (XP-CLR = 122.14) in fatty acid transport (GO: 0015908); ACSL6 (P = 0.0094; XP-EHH = 4.14) and EPHX2 (XP-CLR = 96.97) in fatty acid metabolic process (GO: 0006631). CD36 is a principal skeletal muscle fatty acid transporter, and the mRNA abundance of this gene showed a strong positive correlation with intramuscular fat content, an important component of traits that influence meat quality [42].

**Figure 2:**
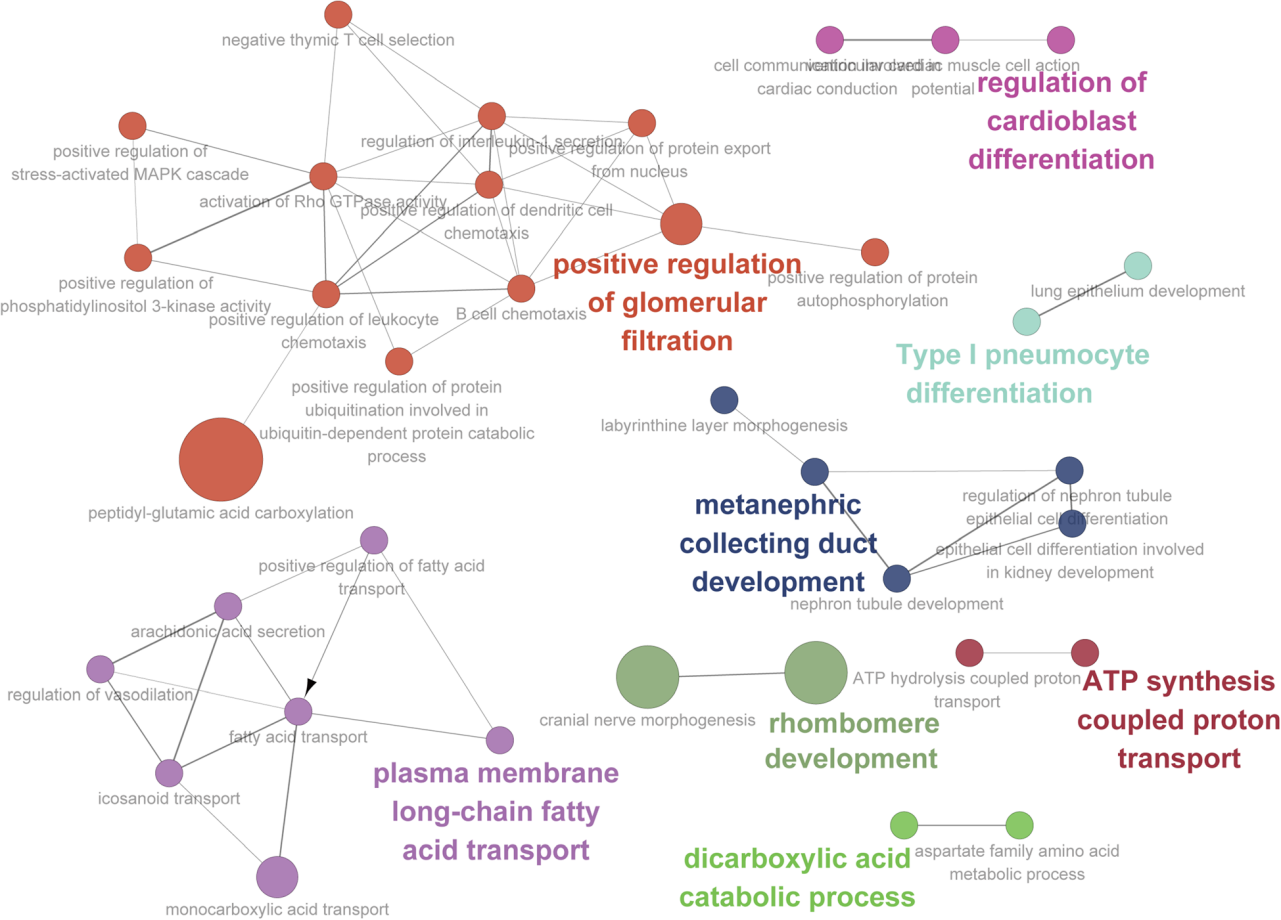
**Gene ontology analysis of 392 putatively advantageous genes in JBP.** Nodes represent gene ontology terms and imply that two gene ontology terms share genes from the considered dataset. The most prominent gene ontology term for each group is highlighted in colors.

In a previous study, genes in the PPAR signaling pathway were significantly associated with traits of porcine meat quality, and KEGG pathway analysis identified two genes enriched in this pathway (*CD36* and *ACSL6*) [43]. Especially, long-chain acyl-CoA synthetase (ACSL) plays an essential role in both lipid biosynthesis and fatty acid degradation, and one of its subfamilies (*ACSL4*) is known for its association with growth and meat quality traits [44]. These candidate genes together may have contributed to the change in fatty acid composition and to the unique features of meat quality in JBP. To further determine biological process at play, we used ClueGO, which integrates gene ontology (GO) categories and creates a functionally organized GO category networks based on the overlap between the different GO categories [26]. The network showed the prominent gene ontology term ‘plasma membrane long-chain fatty acid transport’ as enriched, which may have contributed to the change in fatty acid composition and to the unique features of meat quality in JBP (Figure 2).

### Genes affecting height or body size and strong disease tolerance

Korean native pigs show a slower growth rate and lighter carcass weight [2]. ACE or angiotensin-converting enzyme (XP-CLR = 122.14) inhibitors have been reported to reduce body weight in humans and mice [45,46]. We identified the genes known to be critical for human growth and height from the online Mendelian Inheritance in Man OMIM disease database [47]. The genes which intersected with our selection scan include: *ADCY3* (P = 0.0005; XP-EHH = 5.12), *DNMT3A* (P = 0.0078; XP-EHH = 3.36), *DNAJC27* (P = 0.0085; XP-CLR = 4.20; XP-CLR = 314.97), *DTNB* (P = 0.0044; XP-EHH = 4.59; XP-CLR = 144.73), *PPIL6, ZBTB24,* and *SMPD2* (XP-CLR = 114.20). We also looked for genes related to immune system among genes predicted to be under positive selection in JBP as they exhibit abilities of strong disease tolerance [1]. There was a significant overrepresentation of genes related to ‘positive regulation of immune response’ from XP-CLR scan (GO:0050778, P = 0.036). Animal host defense mechanisms have been a function of the immune system, which aims to detect and eliminate invading pathogens [48]. *ATP6V1H* (XP-CLR = 90.84) is related to defense response to virus (GO: 0051607); *DEFB1* (P = 0.0048; XP-EHH = 4.24) and *TLR3* (P = 0.0028; XP-EHH = 4.54) are involved in defense response to bacterium (GO: 0042742).

### Haplotype analysis of candidate gene region

To further examine the putatively advantageous genes, we analyzed extreme patterns of haplotype differentiation by performing haplotype analyses (Additional file 1: Figure S4). JBP appears to exhibit longer LD patterns and stronger LD blocks in *CACNA1I* and *ZBTB24* gene regions. This suggested that an inherited functional constraint was present in this region; thus, they were retained in JBP through selective sweep from their ancestor.

### Allele frequency threshold analysis and Tajima’s D

**Figure 3:**
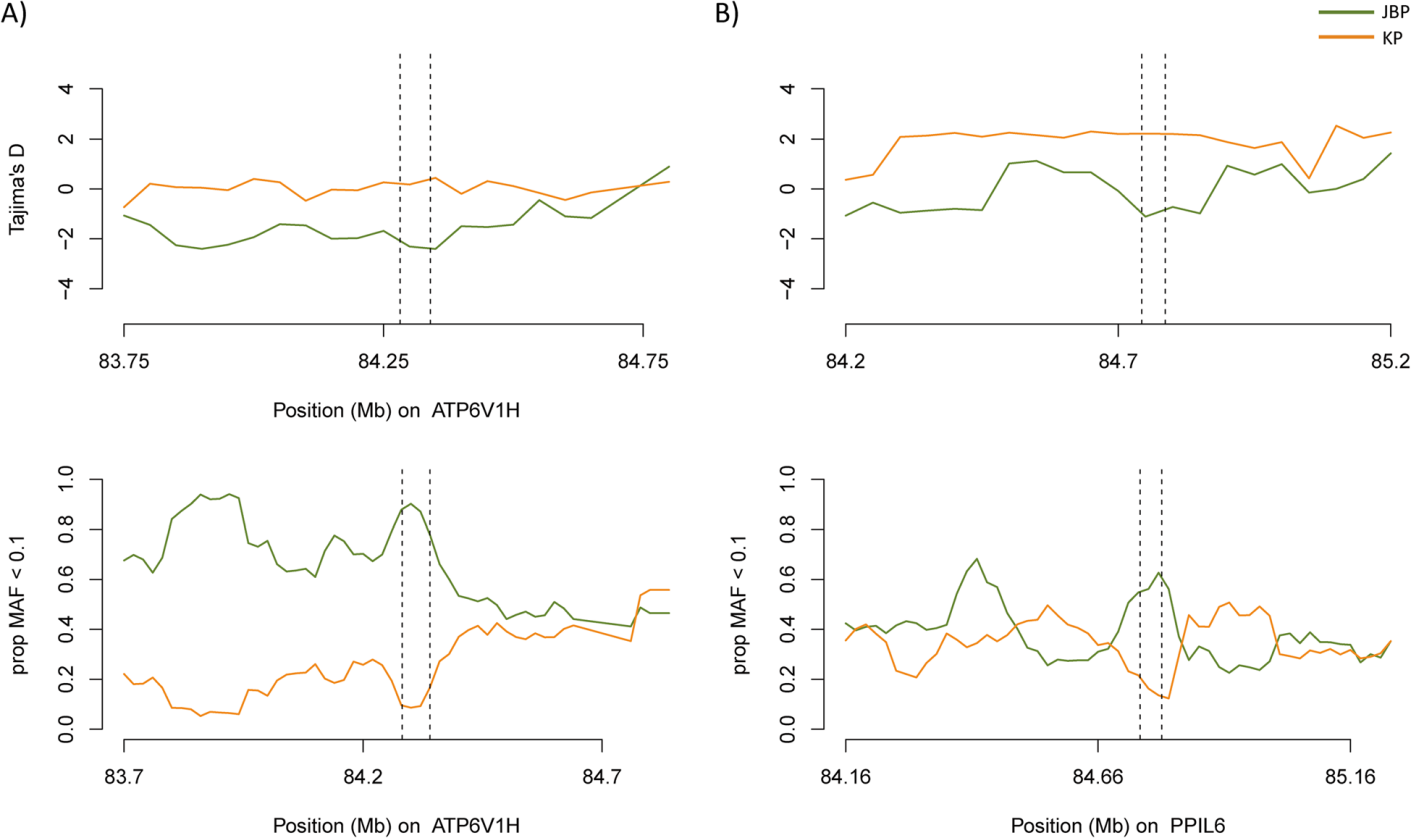
**Minor allele frequency (top) and Tajima’s D analyses of** ***ATP5V1H*** **(A) and** ***PPIL6*** **(B) gene regions.** Plotted is the proportion of SNPs with MAF < 0.10 within 100-kb sliding windows separated by 20-kb steps in Jeju native black pigs (green) and Korean pigs (orange). The vertical dashed bar represents the candidate gene region of each candidate. In the same genomic region, Tajima’s D values in every 50 kb window were plotted for both populations.

The distribution of minor allele frequencies (MAF) around a given genomic region can also suggest particular selective pressures acting on it. An excess of low-frequency alleles could reflect a recent selective sweep [20]. The proportion of SNPs with allele frequencies lower than a threshold (MAF < 0.10) was calculated within sliding windows of 100 kb in size every 20 kb and plotted against physical distance. We focused our attention to the regions around the 9 major candidate genes defined from positive selection scan that intersected with previous functional reports to validate the results. The proportion of SNPs with MAF < 0.10 was plotted within multiple 100-kb sliding windows along 1-Mb regions centered on each major candidate gene for each population. Among genes of interest, the distributions of *ATP6V1H* and *PPIL6* genes in JBP showed an excess of rare alleles within the genic region compared to that in KP population (Figure 3 and Additional file 1: Figure S5).

In addition, analysis using Tajima’s D test also showed significant departure from neutrality and indicated the selective maintenance of alleles within the JBP population compared to KP. Negative values of Tajima’s D indicate an excess of rare variation, consistent with either population growth or positive selection, and we observed a rapid drop of Tajima’s D value within regions of candidate gene under selection in JBP (Figure 3 and Additional file 1: Figure S6).

## Conclusions

JBP offer a rare opportunity to investigate the true genetic resource of once endangered KNBP. Many candidate genes putatively under positive selection were identified, some of which could be crucial for understanding their unique characteristics. Further genome-wide analyses of JBP on a population scale may help conserve and improve native pig resources. Furthermore, as the pig is an exceptional biomedical model related to energy metabolism and obesity in humans, analyzing the genetic basis of native pig breeds may be extended to characterize the effect of putative candidate genes for human [49].

### Availability of supporting data

The whole genome sequence has been deposited at GenBank under the Bioproject accession PRJNA254936.

## Additional files

Additional file 1: Table S1. Summary of resequencing statistics. **Table S2.** Number of SNPs for each chromosome. **Figure S1.** Distribution of SNPs along the genome. **Figure S2.** Distribution plots of XP-EHH raw score. **Figure S3.** Distribution of Minor Allele Frequency (MAF) along the genome. **Figure S4.** Haploview representation of pairwise linkage disequilibria at the *CACNA1I* and *ZBTB24* gene locus in JBP (above) and KP (below) populations. Colors represent *D’* values: dark red = high inter-SNP *D’*; blue = statistically ambiguous *D’*; white – low-inter-SNP *D’*. **Figure S5.** Minor allele frequency analysis of the candidate genes in JBP (green) and KP (red) populations. **Figure S6.** Tajima’s D analysis of the candidate genes in JBP (green) and KP (red) populations. Additional file 2: Table S3. Summary of XP-EHH. Additional file 3: Table S4. Summary of XP-CLR.
