# Supplementary material for: Genome-wide detection and characterization of positive selection in Korean Native Black Pig from Jeju Island
Source: BMC Genet. 2015 Jan 30;16(1):3. doi: 10.1186/s12863-014-0160-1 (PMC4314801; doi:10.1186/s12863-014-0160-1)

**Genome-wide detection and characterization of positive selection in Korean Native Black Pig from Jeju Island**

Authors: Jaemin Kim, Seoae Cho, Kelsey Caetano-Anolles, Heebal Kim, Youn-Chul Ryu

| **SUPPLEMENTARY INFORMATION** |
| --- |

1. **Supplementary Tables**

**Supplementary Table 1…………………………………………………….. 2**

**Supplementary Table 2…………………………………………………….. 3**

1. **Supplementary Figures**

**Supplementary Figure 1…………………………………………………… 4**

**Supplementary Figure 2…………………………………………………… 5**

**Supplementary Figure 3…………………………………………………… 6**

**Supplementary Figure 4…………………………………………………… 7**

**Supplementary Figure 5…………………………………………………… 8**

**Supplementary Figure 6…………………………………………………… 9**

Supplementary Table S1. Summary of resequencing statistics

| Sample  ID | DNA  Sequenced (bp) | Read Depth | Genome Coverage (%) |
| --- | --- | --- | --- |
| 10_453 | 34,064,577,362 | 14.66 | 98.97 |
| 10_561 | 22,971,600,902 | 9.89 | 98.76 |
| 12_98 | 34,260,081,906 | 14.74 | 98.96 |
| K8_17 | 36,144,754,624 | 15.56 | 99.08 |
| KK1 | 31,638,979,962 | 12.56 | 98.35 |
| KK2 | 36,631,567,674 | 14.54 | 98.49 |
| KK3 | 36,036,077,075 | 14.31 | 98.56 |
| KK4 | 40,565,629,709 | 16.1 | 98.50 |
| KK5 | 34,353,068,664 | 13.64 | 98.43 |
| KK6 | 34,090,842,311 | 13.53 | 98.36 |
| KK8 | 35,228,862,135 | 13.99 | 98.53 |
| KK9 | 42,763,646,742 | 16.98 | 98.56 |
| KK10 | 38,048,976,506 | 15.1 | 98.32 |
| KK11 | 35,412,469,474 | 14.06 | 98.52 |

Supplementary Table S2. Number of SNPs for each chromosome.

| Chromosome | Length | SNPs | Change Rate |
| --- | --- | --- | --- |
| 1 | 315,321,322 | 1,486,308 | 212 |
| 2 | 162,569,375 | 1,080,708 | 150 |
| 3 | 144,787,322 | 970,437 | 149 |
| 4 | 143,465,943 | 870,916 | 165 |
| 5 | 111,506,441 | 740,881 | 151 |
| 6 | 157,765,593 | 987,707 | 160 |
| 7 | 134,764,511 | 901,200 | 150 |
| 8 | 148,491,826 | 936,522 | 159 |
| 9 | 153,670,197 | 1,011,102 | 152 |
| 10 | 79,102,373 | 712,943 | 111 |
| 11 | 87,690,581 | 647,008 | 136 |
| 12 | 63,588,571 | 458,054 | 139 |
| 13 | 218,635,234 | 1,155,981 | 189 |
| 14 | 153,851,969 | 847,093 | 182 |
| 15 | 157,681,621 | 832,455 | 189 |
| 16 | 86,898,991 | 704,222 | 123 |
| 17 | 69,701,581 | 534,698 | 130 |
| 18 | 61,220,071 | 446,245 | 137 |
| X | 144,288,218 | 578,854 | 249 |
| Y | 1,637,716 | 2,785 | 588 |
| Total | 2,596,639,456 | 15,906,119 | 163 |

Supplementary Figure S1. Distribution of SNPs along the genome


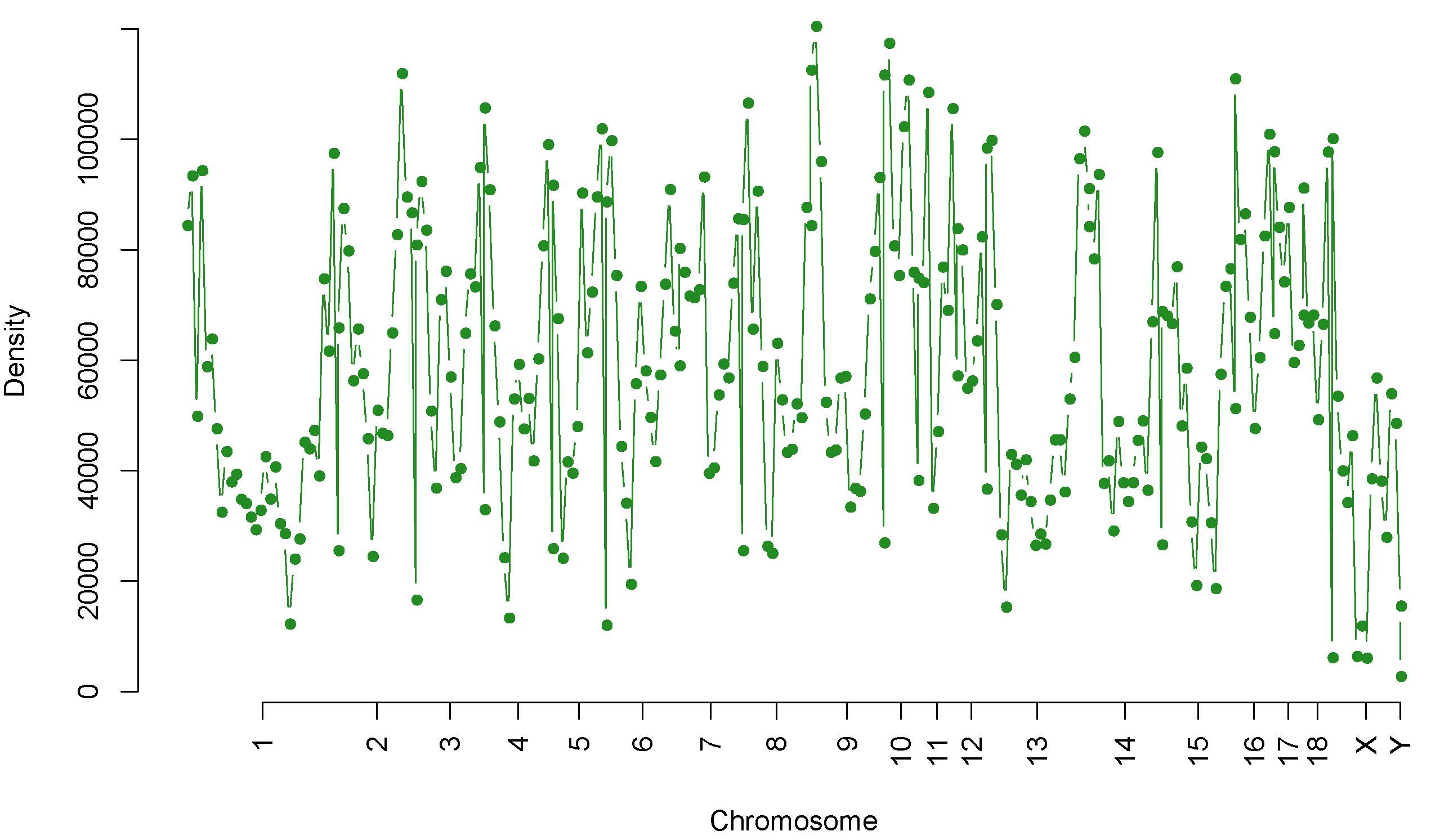


Supplementary Figure S2. Distribution plots of XP-EHH raw score.


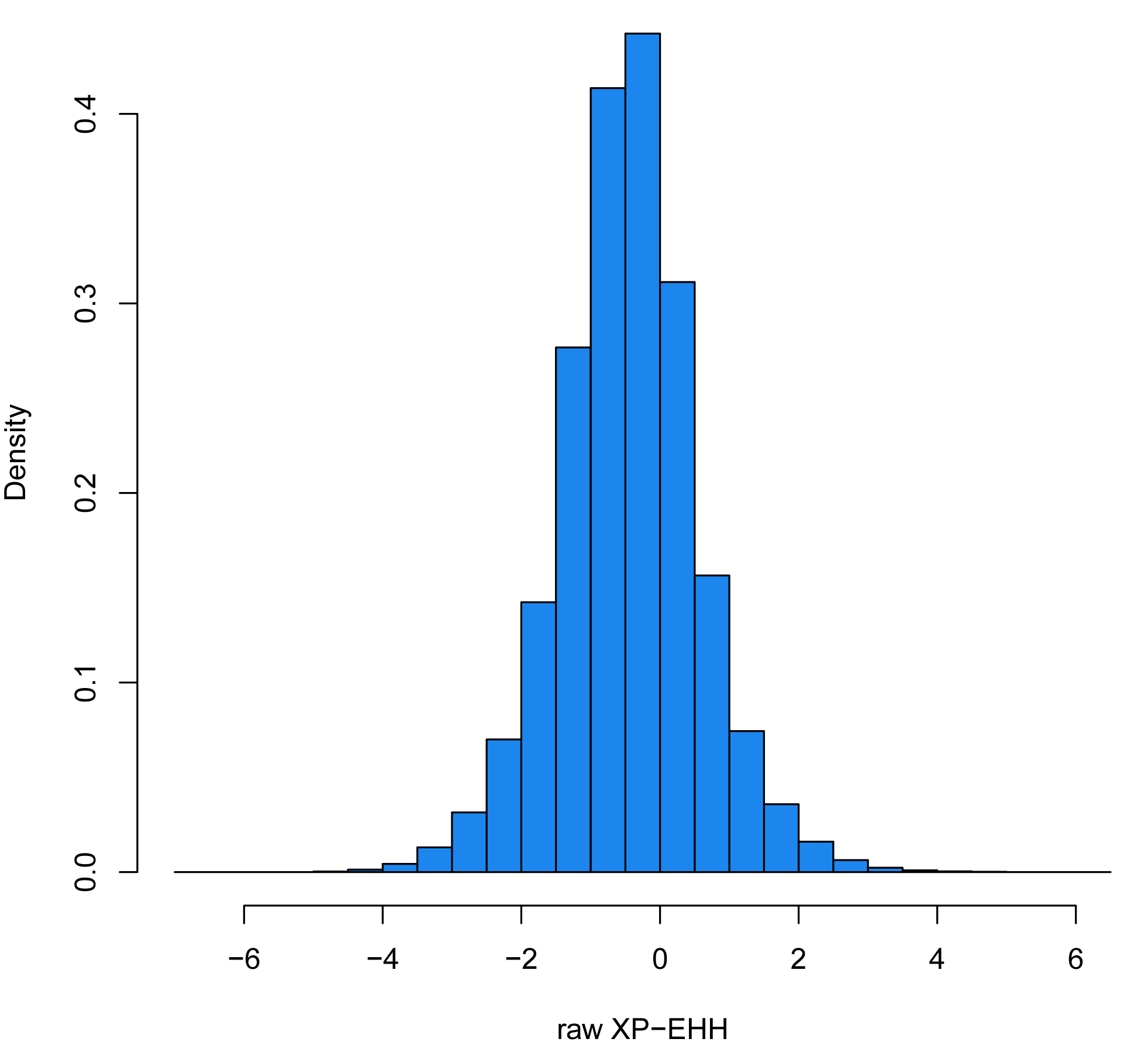


Supplementary Figure S3. Distribution of minor allele frequency (MAF) along the genome.


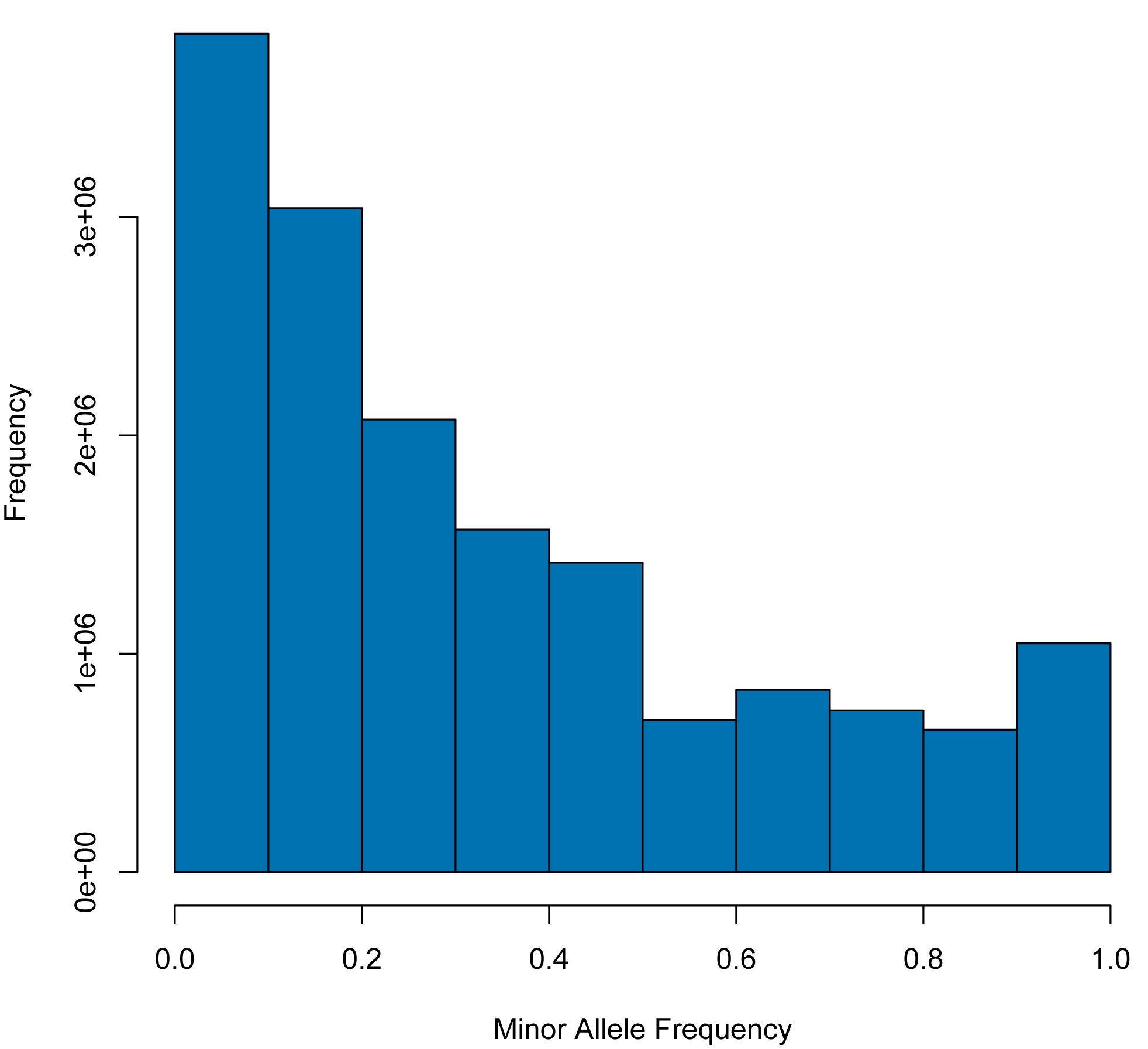


Supplementary Figure S4. Haploview representation of pairwise linkage disequilibria at the *CACNA1I* and *ZBTB24* gene locus in JBP (above) and KP (below) populations. Colors represent *D’* values: dark red = high inter-SNP *D’*; blue = statistically ambiguous *D’*; white – low-inter-SNP *D’*.


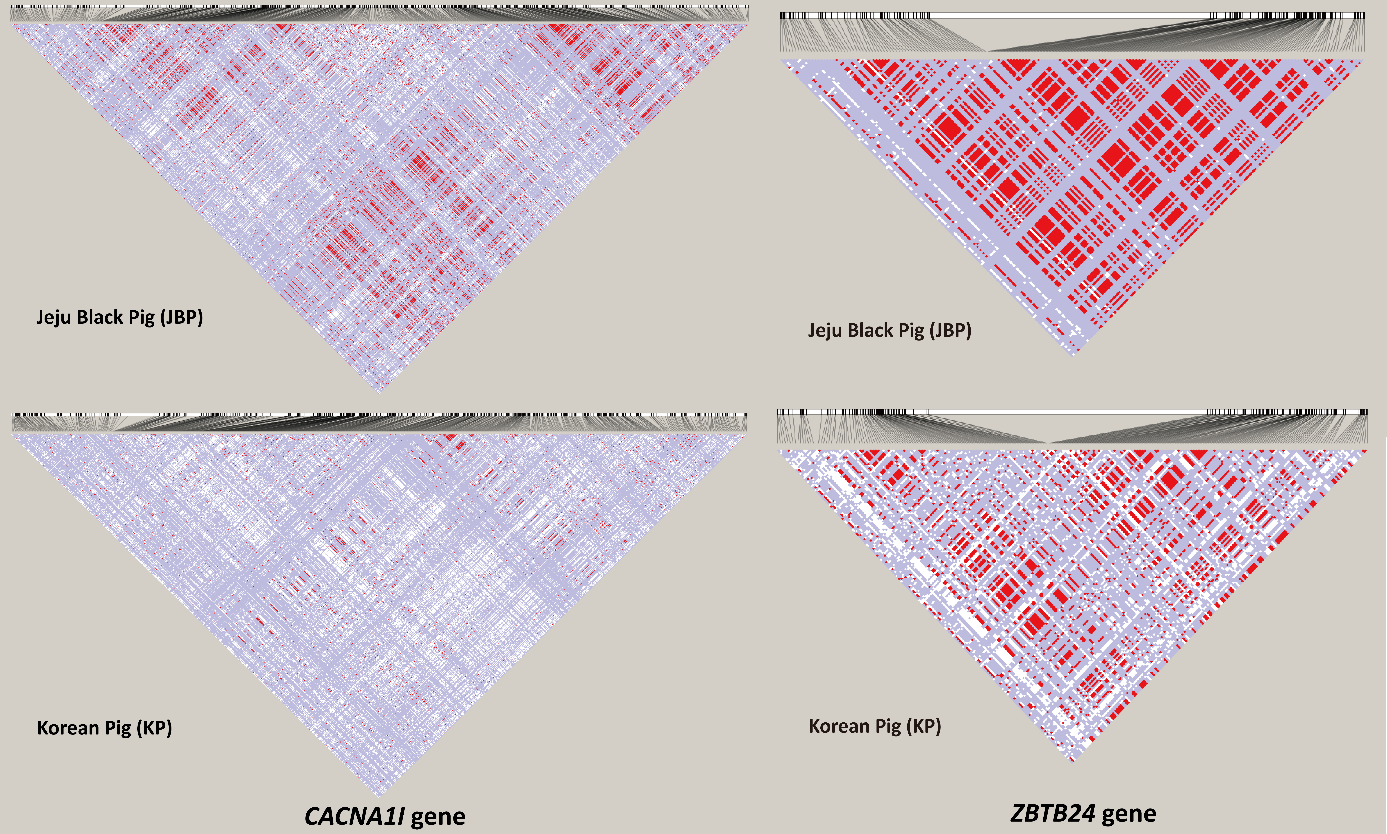


Supplementary Figure S5. Minor allele frequency analysis of the candidate genes in JBP (green) and KP (orange) populations.


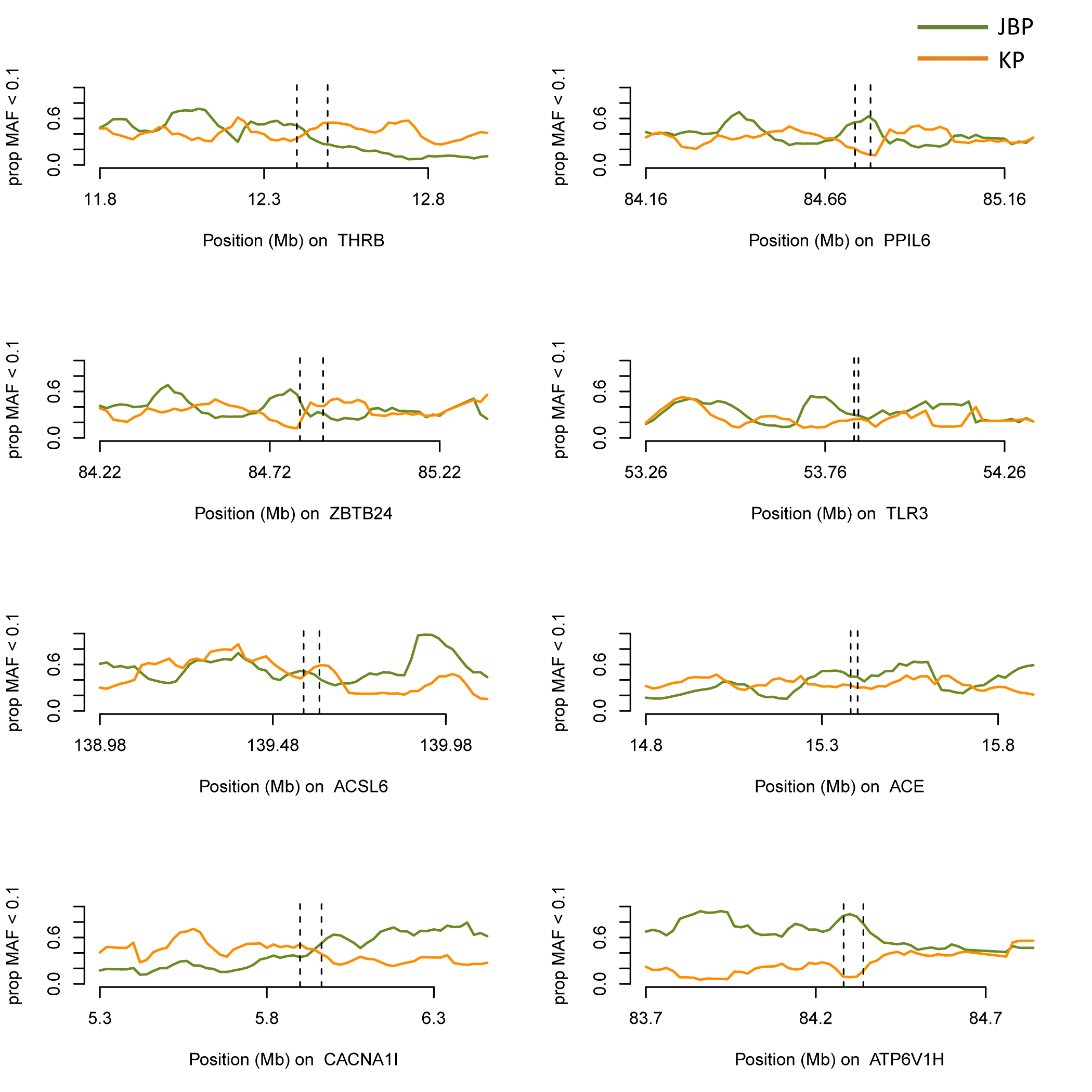


Supplementary Figure S6. Tajima’s D analysis of the candidate genes in JBP (green) and KP (orange) populations.


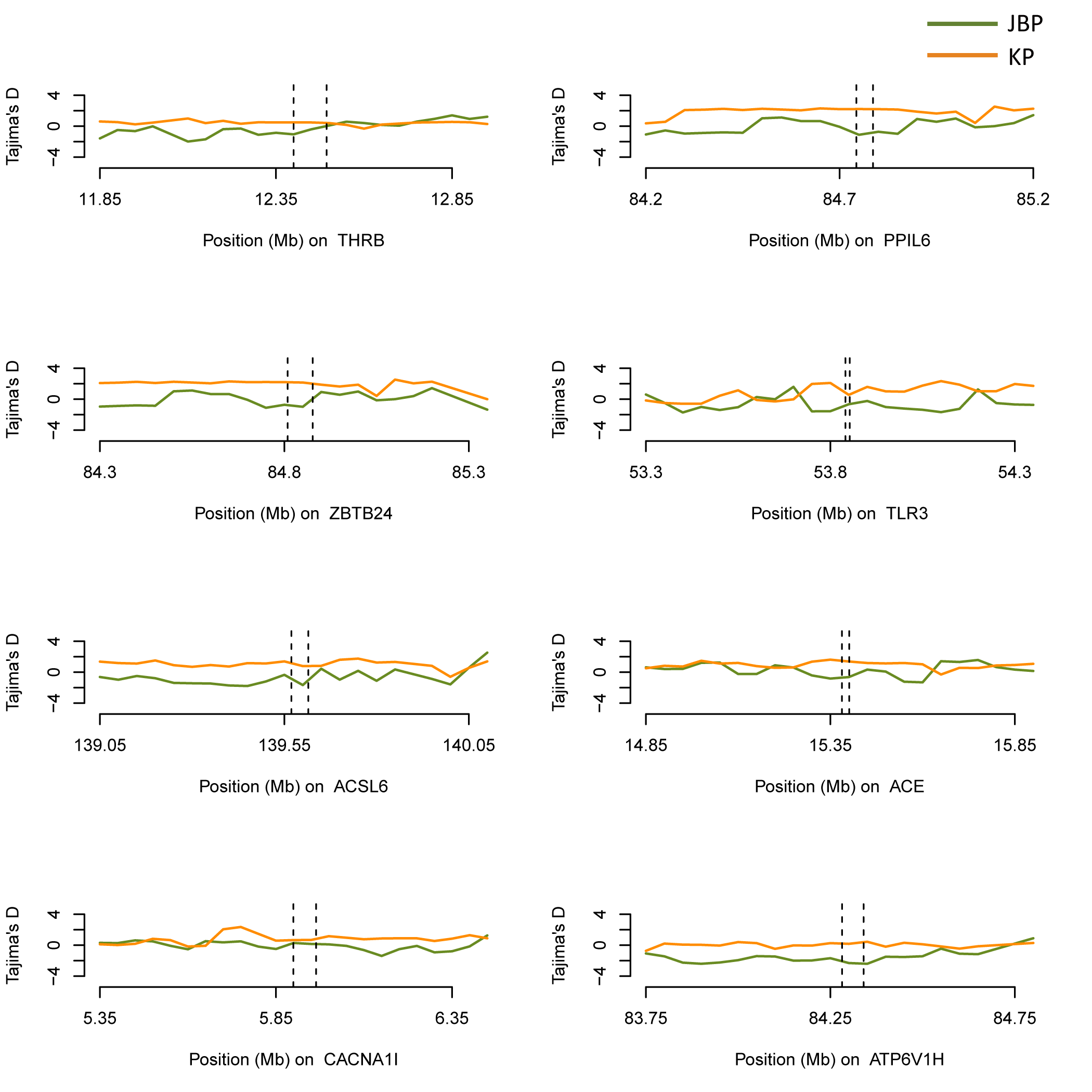

Supplement: Additional file 1: Table S1. — Summary of resequencing statistics. Table S2. Number of SNPs for each chromosome. Figure S1. Distribution of SNPs along the genome. Figure S2. Distribution plots of XP-EHH raw score. Figure S3. Distribution of Minor Allele Frequency (MAF) along the genome. Figure S4. Haploview representation of pairwise linkage disequilibria at the CACNA1I and ZBTB24 gene locus in JBP (above) and KP (below) populations. Colors represent D’ values: dark red = high inter-SNP D’; blue = statistically ambiguous D’; white – low-inter-SNP D’. Figure S5. Minor allele frequency analysis of the candidate genes in JBP (green) and KP (red) populations. Figure S6. Tajima’s D analysis of the candidate genes in JBP (green) and KP (red) populations. [file 12863_2014_160_MOESM1_ESM.docx]
